# Antimicrobial drug use and the risk of glioma: A case–control study

**DOI:** 10.1002/cam4.5222

**Published:** 2022-09-06

**Authors:** Tareq M. Haedenkamp, Michael F. Leitzmann, Ralf A. Linker, Christoph Meier, Claudia Becker, Susan Jick, Peter Hau, Corinna Seliger

**Affiliations:** ^1^ Wilhelm Sander‐NeuroOncology Unit and Department of Neurology Regensburg University Hospital Regensburg Germany; ^2^ Institute of Epidemiology and Preventive Medicine Regensburg University Hospital Regensburg Germany; ^3^ Basel Pharmacoepidemiology Unit, Division of Clinical Pharmacy and Epidemiology, Department of Pharmaceutical Sciences University of Basel Basel Switzerland; ^4^ Boston Collaborative Drug Surveillance Program Lexington MA USA; ^5^ Hospital Pharmacy, University Hospital Basel Basel Switzerland; ^6^ Boston University School of Public Health Lexington MA USA; ^7^ Department of Neurology Heidelberg University Hospital Heidelberg Germany

**Keywords:** antibiotics, antifungals, case–control study, glioma, infectious disease

## Abstract

**Background:**

The use of antibiotics has been associated with increased risks of various cancers. Comprehensive information on the association of antibiotic use with the risk of glioma is lacking.

**Methods:**

We performed a large case–control study based on the Clinical Practice Research Datalink (CPRD) GOLD from the United Kingdom. We identified 4423 glioma cases recorded between 1995 and 2020 and matched them to controls (1:10) on the date of diagnosis (i.e., the index date), age, sex, general practice, and number of years of medical history in the database prior to the index date. We conducted conditional logistic regression analyses to calculate odds ratios (ORs) with 95% confidence intervals (CIs). The exposures of interest were the use of antimicrobial drugs, including antibacterial, antiviral, antifungal, antiprotozoal, and anthelmintic drugs with specific subclasses, where possible.

**Results:**

We found no substantially increased risk of glioma after ever‐use of antibiotics (OR 1.13, 95% CI 1.03–1.24). The risk did not increase with the increasing number of prescriptions received or with increasing time from first use to cancer diagnosis. The use of polyenes was associated with a weakly decreased risk of glioma (OR 0.81, 95% CI 0.67–0.96).

## Graphical abstract

In this large case–control study, we found no substantially increased risk of glioma among users of most antimicrobial drugs. The risk did not increase with increasing numbers of prescriptions received or with increasing time from first use to cancer diagnosis. The use of polyenes yielded a weak inverse association with the risk of glioma, which needs to be confirmed independently.

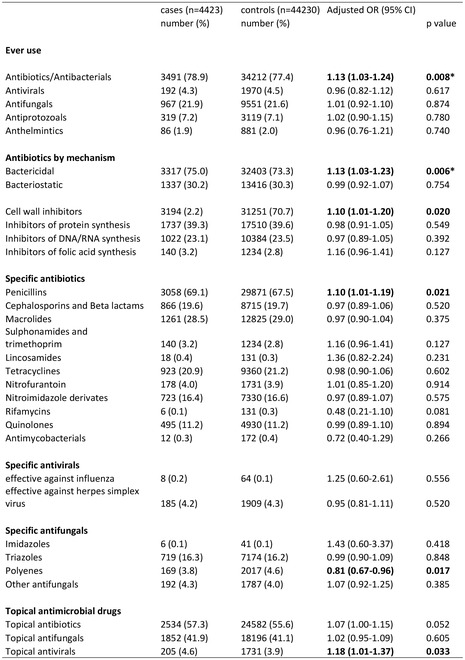

## 1 INTRODUCTION

Gliomas are primary brain tumors with a mostly malignant phenotype.^1^ Glioblastoma is the most common type of glioma.^2^ Glioblastomas are associated with a poor median survival of 15–26 months despite standard therapy with resection, combined radio‐chemotherapy and adjuvant chemotherapy with or without tumor‐treating fields.^3^, ^4^, ^5^ Ionizing radiation is currently the only known environmental factor associated with increased risk of gliomas.^6^

Antibiotics are among the most commonly prescribed drugs, with around 30% of patients receiving at least one antibiotic prescription per year.^7^ Several studies have investigated the association between antibiotic use and risk of different cancers. While some studies have reported an increased risk of several or specific cancers^8^, ^9^, ^10^, ^11^, ^12^, ^13^, ^14^, ^15^, ^16^ in relation to antibiotic use, other investigations found no association,^17^, ^18^, ^19^, ^20^ or no dose‐dependent association.^21^, ^22^ One study observed an inverse association between use of antibiotics and risk of cervical carcinoma.^23^ Only one cohort study based on the Population Registry in Finland reported on the risk of central nervous system (CNS) cancers after antibiotic use and found a slightly increased risk (OR 1.31 after 6 or more prescriptions).^11^

Antibiotics may influence glioma risk through direct or indirect mechanisms. Direct mechanisms include effects of antibiotics on cancer stem cells or differentiated tumor cells such as alteration of mitochondrial ribosome function, as reported for macrolides or tetracyclines.^24^ Indirect mechanisms include long‐term alterations of the intestinal microbiota,^25^ possibly inducing effects on the immune system of the host.^26^ The same pathophysiological considerations apply to other antimicrobials like antifungals, antiprotozoals, or antivirals. For example, infection with herpes simplex virus and treatment with acyclovir independently caused dysbiosis in an animal model.^27^ Since there is no comprehensive information on the association of antimicrobial drug use with the risk of glioma, we performed a case–control study based on data from a large and validated database.

## 2 METHODS

### 2.1 Data source

The data for the current study were obtained from Clinical Practice Research Datalink (CPRD) GOLD. The CPRD is a primary care database with longitudinal medical information on about 11 million patients from more than 670 general practices, representative of the United Kingdom (UK) population with respect to age, sex, and ethnicity.^28^ Data on demographic information, prescriptions, clinical events, and patient characteristics are collected by general practitioners and made available for research.^28^ Previous studies conducted with the CPRD have shown a high validity of the diagnostic coding system.^29^

This study was approved by the Independent Scientific Advisory Committee for Medicines and Healthcare products Regulatory Agency database research (protocol no: 19_189).

The study protocol was made available to journal editors and peer reviewers (additional file for review but not for publication).

Data for this study were derived from CPRD primary care data obtained under license from the UK Medicines and Healthcare products Regulatory Agency. The data are provided by patients and collected by the National Health Service (NHS) as part of their care and support. The interpretation and conclusions contained in this study are those of the authors alone.

### 2.2 Cases

Cases were all patients aged less than 90 years at first glioma diagnosis between 1995 and 2020. We used Read codes to identify patients with an incident diagnosis of glioma (see Table S1). The index date was defined as the date of the first glioma diagnosis minus 1 year to account for the lag time between tumor development and detection. In order to include only incident glioma cases and to ensure enough history in the database to ascertain exposure, we included only patients with at least 3 years of active history in the database before the index date. We excluded all patients with a history of any other cancer, recorded alcoholism or human immunodeficiency virus infection prior to the index date.

### 2.3 Controls

We matched 10 controls to each case on index date, age (plus/minus 2 years), sex, general practice (where it was possible), and number of years of medical history in the database prior to the index date (plus/minus 2 years). We applied the same exclusion criteria to controls as to cases. Furthermore, patients with a history of craniotomy in the year before the index date were not eligible to be controls in order to minimize the risk of using control patients with unrecorded glioma diagnosis. We described a similar approach for identification of cases and controls in a previous paper.^30^, ^31^

### 2.4 Exposures

In this study, the term “antibiotic” refers to antibacterial drugs specifically. The term antimicrobial drugs refer to the entire group of antibacterial, antiviral, antifungal, antiprotozoal, and anthelmintic drugs.

The use of antibiotic drugs, defined as at least 1 recorded prescription for an antibiotic drug, was the exposure of interest in this study. First, we assessed antibiotic use as a combined variable for any antibiotic drug, and then we investigated the following common antibiotic classes separately: penicillins, cephalosporins, and other beta‐lactams, chloramphenicol, macrolides, aminoglycosides, glycopeptides, sulfonamides and trimethoprim, linezolid, lipopeptides, lincosamides, tetracyclines, nitrofurantoin, nitroimidazole derivates, quinolones, rifamycins, drugs against mycobacteria, and lastly a group of other remaining antibiotics. Additionally, we classified antibiotics by mechanism (inhibitors of cell wall synthesis, protein synthesis, deoxyribonucleic acid (DNA) and ribonucleic acid (RNA) synthesis, folic acid synthesis) and by bactericidal versus bacteriostatic action. We also explored exposure to antiviral, antifungal, antiprotozoal, anthelmintic drugs, and topical antimicrobial drugs, and to specific subgroups of drugs (antivirals against herpes simplex virus, hepatitis viruses, human immunodeficiency virus and influenza, echinocandins, imidazoles, triazoles, polyenes, and other antifungals).

We defined ever‐use of antibiotics as one or more antibiotic prescriptions before the index date. We further examined the dose‐dependency of the association by exploring the number of prescriptions before the index date (1 prescription, 2–4 prescriptions, ≥5 prescriptions). Cut‐off values were primarily based on quartiles and median value of number of prescriptions of any antimicrobial drug and adjusted because of small case numbers in the category with most prescriptions. We also explored time since first prescription (1–5 years, 6–10 years, 11–15 years, >15 years).

Finally, we assessed the association between history of infectious disease and the risk of glioma. We considered infections of the central nervous system, pneumonia, other infections of the respiratory tract, sepsis, fungal infections, gastrointestinal infections, hepatic and biliary infections, urogenital infections, malaria, tuberculosis, syphilis, and skin infections as specific exposures.

### 2.5 Statistical analysis

We conducted conditional logistic regression analyses to calculate odds ratios (ORs) with 95% confidence intervals (CIs).

In univariate analyses, we investigated the influence of potential confounding variables including body mass index (BMI) (closest recording prior to the index date), smoking status (closest recording prior to the index date), common comorbidities (asthma, chronic obstructive lung disease (COPD), arrhythmia, congestive heart failure, myocardial infarction, hypertension, stroke, hyperlipidemia, diabetes, deep vein thrombosis (DVT), epilepsy, renal disease) and use of nonsteroidal anti‐inflammatory drugs (NSAIDs), opioids, corticosteroids, and immunosuppressive drugs. We assumed that patients with comorbidities and those under treatment with corticosteroids or other immunosuppressive drugs tend to have more infections. We only included variables that significantly altered the beta‐estimate of the risk estimate of glioma by >10% in the final multivariable analysis. Infectious diseases were included in the multivariable model a priori, irrespective of the results of the univariate analysis.

We stratified all analyses by sex, age group (<40, 40–60, >60 years), and glioma subtype.

We also conducted tests of linear trend by modeling the median value of each category of the number of antibiotic prescriptions, the time since first prescription, and the duration of exposure as a continuous variable in the multivariable model, the coefficient for which was evaluated using a Wald test.

We considered a two‐sided *p*‐value of <0.05 as statistically significant. We corrected p value thresholds of the analyses of specific antibiotic drugs for multiple testing according to the Bonferroni method. We performed those corrections separately for the main antimicrobial classes, classifications by mechanism, and specific antibiotics because the drugs were the same in each of these categories.

We used SAS version 9.4 (SAS Institute Inc) to perform statistical analyses.

## 3 RESULTS

We identified 4423 glioma cases and 44,230 matched controls, with a mean (SD) age of 54 (±19.54) years and with slightly more men (55.1%) than women (44.9%). Matching on general practice was possible for 42,789 controls (96.7%). Cases had a mean history in the database of 11.8 years prior to the index date. A total of 880 cases (19.9%) had a World Health Organization grade I/II/III glioma, 1910 cases (43.2%) had glioblastoma, and 1633 cases (36.9%) had a glioma that was not further specified. More information on basic characteristics of cases and controls is depicted in Table 1. In accordance with the CPRD guidelines, we show no results that contain less than five events.

**TABLE 1:** Patient characteristics of glioma cases and their matched controls

		Cases (*n* = 4423)	Controls (*n* = 44,230)	Crude OR (95% CI)	*p* value
		Number (%)	Number (%)		
Sex					
Male		2437 (55.1)	24,370 (55.1)		
Female		1986 (44.9)	19,860 (44.9)		
Age group					
0–39		930 (21.0)	9279 (21.0)		
40–59		1404 (31.7)	14,042 (31.8)		
≥60		2089 (47.2)	20,909 (47.3)		
Number of recorded years prior to the index date					
≤5		519 (11.7)	5138 (11.6)		
6–10		1482 (33.5)	14,608 (33.0)		
11–20		1922 (43.5)	19,481 (44.0)		
>20		500 (11.3)	5003 (11.3)		
Smoking status					
Smoker		662 (15.0)	7320 (16.6)	**0.84 (0.77–0.92)**	<**0.001**
Past‐smoker		1093 (24.7)	10,784 (24.4)	0.96 (0.88–1.04)	0.296
Non‐smoker		2062 (46.6)	19,348 (43.7)	1.00 (reference)	
Unknown		606 (13.7)	6778 (15.3)	**0.74 (0.65–0.84)**	<**0.001**
BMI (kg/m^2^)					
<18.5		29 (0.7)	553 (1.3)	**0.52 (0.36–0.76)**	**0.001**
18.5–24.9		1222 (27.6)	12,159 (27.5)	1.00 (reference)	
25–29.9		1315 (29.7)	12,623 (28.5)	1.04 (0.96–1.13)	0.361
≥30		772 (17.5)	7748 (17.5)	0.99 (0.90–1.10)	0.910
Unknown		1085 (24.5)	11,147 (25.2)	0.95 (0.85–1.05)	0.286
Comorbidities					
Asthma		571 (12.9)	5823 (13.2)	0.98 (0.89–1.07)	0.624
COPD		110 (2.5)	1320 (3.0)	0.82 (0.67–1.01)	0.051
Arrhythmia		125 (2.8)	1176 (2.7)	1.07 (0.88–1.29)	0.509
Congestive heart failure		51 (1.2)	722 (1.6)	**0.70 (0.52–0.93)**	**0.010**
Myocardial infarction		120 (2.7)	1497 (3.4)	**0.79 (0.65–0.96)**	**0.012**
Hypertension		1065 (24.1)	10,905 (24.7)	0.96 (0.89–1.04)	0.341
Stroke		146 (3.3)	1684 (3.8)	0.86 (0.72–1.02)	0.078
Hyperlipidemia		450 (10.2)	4726 (10.7)	0.94 (0.84–1.05)	0.258
Diabetes		270 (6.1)	3084 (7.0)	**0.86 (0.75–0.98)**	**0.023**
DVT		86 (1.9)	616 (1.4)	**1.41 (1.12–1.77)**	**0.005**
Epilepsy		206 (4.7)	712 (1.6)	**3.04 (2.59–3.57)**	<**0.001**
Renal disease		130 (2.9)	1075 (2.4)	**1.22 (1.01–1.47)**	**0.036**
Comedication	Number of prescriptions				
NSAIDs	0	1879 (42.5)	19,275 (43.6)	1.00 (reference)	
	1–9	1982 (44.8)	19,320 (43.7)	1.06 (0.99–1.14)	
	≥10	562 (12.7)	5635 (12.7)	1.03 (0.93–1.15)	
Opioids	0	2989 (67.6)	30,632 (69.3)	1.00 (reference)	
	1–9	1104 (25.0)	10,192 (23.0)	**1.12 (1.04–1.21)**	
	≥10	330 (7.5)	3406 (7.7)	1.01 (0.89–1.14)	
Corticosteroids	0	3320 (75.1)	33,247 (75.2)	1.00 (reference)	
	1–9	983 (22.2)	9558 (21.6)	1.03 (0.95–1.11)	
	≥10	120 (2.7)	1425 (3.2)	0.84 (0.69–1.02)	
Immunosupressants	0	4378 (99.0)	43,711 (98.8)	1.00 (reference)	
	1–9	11 (0.3)	155 (0.4)	0.71 (0.38–1.31)	
	≥10	34 (0.8)	364 (0.8)	0.93 (0.65–1.33)	

*Note*: No correction for multiple testing was performed.

Abbreviations: BMI, body mass index; COPD, chronic obstructive pulmonary disease; DVT, deep vein thrombosis; NSAID, nonsteroidal anti‐inflammatory drug.

We considered a two‐sided *p* value of <0.05 as statistically significant. These results are indicated in bold.

In univariate analyses, cases were less likely to be smokers than controls (OR 0.84, 95% CI 0.77–0.92). Low BMI (BMI < 18.5) was associated with lower risk of glioma (OR 0.52, 95% CI 0.36–0.76) compared to normal weight. Congestive heart failure (OR 0.70, 95% CI 0.52–0.93), myocardial infarction (OR 0.79, 95% CI 0.65–0.96), and diabetes (OR 0.86, 95% CI 0.75–0.98) were comorbidities with an inverse relation to glioma, whereas DVT (OR 1.41, 95% CI 1.12–1.77), epilepsy (OR 3.04, 95% CI 2.59–3.57) and renal disease (OR 1.22, 95% CI 1.01–1.47) showed an increased risk. Concomitant use of opioids, NSAIDs, corticosteroids, and immunosuppressive drugs had no impact on risk of glioma (Table 1).

When we performed univariable analyses of various infectious diseases to assess their potential as confounders, none of the specific infectious diseases investigated (pneumonia, other respiratory tract infection, fungal infection, gastrointestinal tract infection, hepatitis and biliary tract infection, urogenital infection, and skin infections) were materially related to the risk of developing glioma (Table 2).

**TABLE 2:** Infectious diseases and the risk of glioma

Infectious disease	Cases (*n* = 4423)	Controls (*n* = 44,230)	Crude OR (95% CI)	*p* value
	Number (%)	Number (%)		
Any infection	3038 (68.69)	29,808 (67.39)	**1.08 (1.00–1.17)**	**0.045**
CNS infection	20 (0.5)	162 (0.4)	1.24 (0.78–1.97)	0.386
Pneumonia	94 (2.1)	992 (2.2)	0.95 (0.76–1.17)	0.610
Other respiratory tract infection	2325 (52.6)	23,307 (52.7)	0.99 (0.93–1.07)	0.852
Sepsis	15 (0.3)	141 (0.3)	1.06 (0.62–1.82)	0.820
Fungal infection	362 (8.2)	3594 (8.1)	1.01 (0.90–1.14)	0.885
Gastrointestinal tract infection	500 (11.3)	4728 (10.7)	1.07 (0.97–1.18)	0.199
Hepatitis and biliary tract infection	47 (1.1)	406 (0.9)	1.16 (0.86–1.58)	0.346
Urogenital infection	541 (12.2)	5277 (11.9)	1.03 (0.93–1.15)	0.525
Malaria	7 (0.2)	58 (0.1)	1.21 (0.55–2.64)	0.647
Tuberculosis	40 (0.9)	446 (1.0)	0.90 (0.65–1.24)	0.500
Skin infection and abscess	1420 (32.1)	13,715 (31.0)	1.06 (0.99–1.14)	0.110
By latency (years)				
Pneumonia				
Never	4329 (97.9)	43,241 (97.8)	1.00 (reference)	
<5	20 (0.5)	287 (0.7)	0.70 (0.44–1.10)	
5–10	26 (0.6)	166 (0.4)	**1.56 (1.03–2.36)**	
>10	48 (1.1)	536 (1.2)	0.89 (0.66–1.21)	
*p* value for trend				0.554
Other respiratory tract infection				
Never	2100 (47.5)	20,926 (47.3)	1.00 (reference)	
<5	661 (14.9)	6369 (14.4)	1.04 (0.94–1.14)	
5–10	673 (15.2)	7023 (15.9)	0.95 (0.86–1.05)	
>10	989 (22.4)	9912 (22.4)	0.99 (0.89–1.09)	
*p* value for trend				0.612
Fungal infection				
Never	4061 (91.8)	40,636 (91.9)	1.00 (reference)	
<5	135 (3.1)	1144 (2.6)	1.19 (0.99–1.43)	
5–10	100 (2.3)	1056 (2.4)	0.95 (0.77–1.17)	
>10	127 (2.9)	1394 (3.2)	0.90 (0.74–1.10)	
*p* value for trend				0.996
Gastrointestinal tract infection				
Never	3923 (88.7)	39,505 (89.3)	1.00 (reference)	
<5	163 (3.7)	1570 (3.6)	1.05 (0.89–1.24)	
5–10	147 (3.3)	1377 (3.1)	1.08 (0.91–1.29)	
>10	190 (4.3)	1778 (4.0)	1.08 (0.92–1.27)	
*p* value for trend				0.349
Hepatitis and biliary tract infection				
Never	4376 (98.9)	43,830 (99.1)	1.00 (reference)	
<5	5 (0.1)	57 (0.1)	0.88 (0.35–2.20)	
5–10	7 (0.2)	56 (0.1)	1.26 (0.57–2.78)	
>10	35 (0.8)	287 (0.7)	1.22 (0.86–1.74)	
*p* value for trend				0.215
Urogenital infection				
Never	3882 (87.8)	38,954 (88.1)	1.00 (reference)	
<5	176 (4.0)	1654 (3.7)	1.07 (0.91–1.26)	
5–10	152 (3.4)	1401 (3.2)	1.10 (0.92–1.31)	
>10	213 (4.8)	2221 (5.0)	0.96 (0.82–1.12)	
*p* value for trend				0.243
Skin infection and abscess				
Never	3003 (67.9)	30,518 (69.0)	1.00 (reference)	
<5	539 (12.2)	4982 (11.3)	1.10 (1.00–1.22)	
5–10	426 (9.6)	4684 (10.6)	1.08 (0.96–1.20)	
>10	455 (10.3)	4684 (10.6)	0.99 (0.88–1.11)	
*p* value for trend				0.681

*Note*: Not reportable (<5 events): Syphilis. We performed Bonferroni correction for multiple testing of *p* value thresholds. Significant results after Bonferroni correction are indicated by an asterisk (*).

Abbreviations: CNS, central nervous system.

We considered a two‐sided *p* value of <0.05 as statistically significant. These results are indicated in bold.

We considered comorbidities and co‐medications that were statistically significantly associated with glioma risk as potential confounding factors along with every specific infectious disease. We included the corresponding variables in the multivariable analysis of antimicrobial drug use.

First, we investigated the role of any antimicrobial drug use. Despite reaching statistical significance, the relative risk of glioma for ever‐use of antibacterials, in general, was not substantially increased (OR 1.13, 95% CI 1.03–1.24), nor was there an association with the use of antivirals, antifungals, antiprotozoals, or anthelmintics (Table 3).

**TABLE 3:** Ever‐use of antimicrobial drugs and risk of glioma

Antimicrobial drug	Cases (*n* = 4423)	Controls (*n* = 44,230)	Adjusted OR (95% CI)	*p* value
	Number (%)	Number (%)		
Ever‐use				
Antibiotics/Antibacterials	3491 (78.9)	34,212 (77.4)	**1.13 (1.03–1.24)**	**0.008** *
Antivirals	192 (4.3)	1970 (4.5)	0.96 (0.82–1.12)	0.617
Antifungals	967 (21.9)	9551 (21.6)	1.01 (0.92–1.10)	0.874
Antiprotozoals	319 (7.2)	3119 (7.1)	1.02 (0.90–1.15)	0.780
Anthelmintics	86 (1.9)	881 (2.0)	0.96 (0.76–1.21)	0.740
Antibiotics by mechanism				
Bactericidal	3317 (75.0)	32,403 (73.3)	**1.13 (1.03–1.23)**	**0.006** *
Bacteriostatic	1337 (30.2)	13,416 (30.3)	0.99 (0.92–1.07)	0.754
Cell wall inhibitors	3194 (2.2)	31,251 (70.7)	**1.10 (1.01–1.20)**	**0.020**
Inhibitors of protein synthesis	1737 (39.3)	17,510 (39.6)	0.98 (0.91–1.05)	0.549
Inhibitors of DNA/RNA synthesis	1022 (23.1)	10,384 (23.5)	0.97 (0.89–1.05)	0.392
Inhibitors of folic acid synthesis	140 (3.2)	1234 (2.8)	1.16 (0.96–1.41)	0.127
Specific antibiotics				
Penicillins	3058 (69.1)	29,871 (67.5)	**1.10 (1.01–1.19)**	**0.021**
Cephalosporins and beta lactams	866 (19.6)	8715 (19.7)	0.97 (0.89–1.06)	0.520
Macrolides	1261 (28.5)	12,825 (29.0)	0.97 (0.90–1.04)	0.375
Sulfonamides and trimethoprim	140 (3.2)	1234 (2.8)	1.16 (0.96–1.41)	0.127
Lincosamides	18 (0.4)	131 (0.3)	1.36 (0.82–2.24)	0.231
Tetracyclines	923 (20.9)	9360 (21.2)	0.98 (0.90–1.06)	0.602
Nitrofurantoin	178 (4.0)	1731 (3.9)	1.01 (0.85–1.20)	0.914
Nitroimidazole derivates	723 (16.4)	7330 (16.6)	0.97 (0.89–1.07)	0.575
Rifamycins	6 (0.1)	131 (0.3)	0.48 (0.21–1.10)	0.081
Quinolones	495 (11.2)	4930 (11.2)	0.99 (0.89–1.10)	0.894
Antimycobacterials	12 (0.3)	172 (0.4)	0.72 (0.40–1.29)	0.266
Specific antivirals				
Effective against influenza	8 (0.2)	64 (0.1)	1.25 (0.60–2.61)	0.556
Effective against herpes simplex virus	185 (4.2)	1909 (4.3)	0.95 (0.81–1.11)	0.520
Specific antifungals				
Imidazoles	6 (0.1)	41 (0.1)	1.43 (0.60–3.37)	0.418
Triazoles	719 (16.3)	7174 (16.2)	0.99 (0.90–1.09)	0.848
Polyenes	169 (3.8)	2017 (4.6)	**0.81 (0.67–0.96)**	**0.017**
Other antifungals	192 (4.3)	1787 (4.0)	1.07 (0.92–1.25)	0.385
Topical antimicrobial drugs				
Topical antibiotics	2534 (57.3)	24,582 (55.6)	1.07 (1.00–1.15)	0.052
Topical antifungals	1852 (41.9)	18,196 (41.1)	1.02 (0.95–1.09)	0.605
Topical antivirals	205 (4.6)	1731 (3.9)	**1.18 (1.01–1.37)**	**0.033**

*Note*: Adjusted for: BMI, smoking, diabetes, congestive heart failure, myocardial infarction, deep vein thrombosis, epilepsy, renal disease, opioid use, and infectious diseases. Not reportable (<5 events): Aminoglycosides, chloramphenicol, glycopeptides, linezolid, lipopeptides, other remaining antibiotics, antivirals against hepatitis and human immunodeficiency virus, echinocandins. We performed Bonferroni correction for multiple testing of *p* value thresholds.

* Significant results after Bonferroni correction.

We considered a two‐sided *p* value of <0.05 as statistically significant. These results are indicated in bold.

There was no material interdependence between the use of antibiotic drugs according to mechanism of action and risk of glioma (Table 3).

When we examined specific antimicrobial drugs there was no meaningful relation between ever‐use of penicillins and risk of glioma (OR 1.10, 95% CI 1.01–1.19). By comparison, use of polyenes, which are a specific class of antifungals, yielded an inverse association with the risk of glioma (OR 0.81, 95% CI 0.67–0.96) (Table 3).

Results did not change materially upon stratification by age and glioma subtype. In analyses stratified by sex the Ors for ever‐use of antibiotics, bactericidal drugs, cell wall inhibitors, and penicillins were not materially different in women compared to men. In contrast, the use of folic acid synthesis inhibitors was related to an increased glioma risk in women (OR 1.39, 95% CI 1.07–1.80), but not in men (OR 0.94, 95% CI 0.70–1.26).

There was no association between number of prescriptions for any of the antibiotics or microbials and risk of glioma when we examined the numbers of prescriptions recorded before the index date. (Table S2). Time of first antimicrobial use showed no importance regarding the risk of glioma. For 1 to 5, 6 to 10, 11 to 15, or more than 15 years since first prescription, use of polyenes showed inverse, albeit statistically non‐significant ORs of 0.88, 0.79, 0.69, and 0.75, respectively, but the test for linear trend was statistically significant (*p* value for trend 0.016) (Table S3).

After correction for multiple testing, the only associations that remained statistically (but likely not clinically) significant were those of ever‐use of total antibiotics and bactericidal antibiotics to risk of glioma.

## 4 DISCUSSION

In this large case–control study based on 4423 glioma cases and 44,230 controls, we found no association between the use of antibiotics and a materially altered risk of developing glioma, regardless of type of antibiotic, cumulative dose (number of prescriptions), or timing of use.

While there are many studies that investigated the effect of antibiotic use on other cancers, especially colorectal cancer and breast cancer,^8^, ^10^, ^14^, ^16^, ^18^, ^20^, ^32^ to date, there is no specific information on the association between antibiotic use and the occurrence of gliomas.

A nested case–control study from New Zealand included 6678 patients with any cancer and found positive associations of breast cancer, lung cancer, and colorectal cancer with use of antibiotics. In that study, 95 cases had brain tumors that were not further classified and previous use of antibiotics was not associated with brain tumor risk.^9^ A cohort study based on the Population Registry in Finland examined antibiotic use between 1995 and 1997 in more than 3 million healthy individuals and ascertained 134,070 cancers during follow‐up between 1998 and 2004, 4351 of which were some form of brain tumor. The authors of that study observed an increased risk of brain tumors after antibiotic use.^11^ However, the study did not provide information on the classification of brain tumors. Conclusions from their results are therefore limited, as brain tumors include more than 200 distinct entities and only around 24.5% of brain tumors are gliomas.^2^

Antibiotics may be associated with risk of cancer in several ways: First, a direct mechanism of action is conceivable. Doxycycline, azithromycin, tigecycline, and the antiparasitic drug pyrvinium pamoate inhibit sphere formation in several cancer stem cell lines, presumably by targeting mitochondrial ribosome function.^24^ Penicillins are classical beta‐lactam antibiotics that act through acylation of the active site of the bacterial cell wall transpeptidase.^33^ To our knowledge, there is no information available on direct effects of penicillins on tumor cell growth. Another intriguing link between antibiotic use and glioma risk are mechanisms involving alterations of the intestinal microbiota. We previously reported a positive association between inflammatory bowel diseases, that usually show dysbiosis, and the risk of glioma in patients younger than 40 years.^31^ The half‐life period of most antimicrobial drugs is only a few hours, which raises the question how these drugs could possibly have an impact on the development of gliomas years after their first prescription. It is well documented that antibiotic use leads to long‐lasting perturbations of the microbial diversity in the gut both in humans and animal models.^25^, ^34^, ^35^ A recent meta‐analysis showed that antibiotics including penicillins such as amoxicillin, increase the abundance of enterobacteriacea.^36^ A strong depletion of anaerobic bacteria is observed after use of the penicillin piperacillin.^36^ Numerous antibiotics are associated with a decrease in butyrate‐producing bacteria.^36^ Perinatal administration of beta‐lactams leads to lower serum levels of short chain fatty acids (SCFAs) in infants.^37^ SCFAs play an important role in maturation and function of microglia of the CNS^38^ and could therefore represent a candidate for the interaction of antibiotic‐induced alterations of the intestinal microbiota and glioma risk. Furthermore, alterations of the intestinal microbiota modulate immunological cells not only in the gut, but also in the peripheral blood by mechanisms involving SCFAs,^39^, ^40^ as well as serum cytokine levels.^41^ A dysfunctional immune system plays an important role in the pathophysiology of gliomas.^42^ Apart from SCFAs, penicillins modulate serum levels of other metabolites such as those of serotonine and bile acids.^43^, ^44^ The results of our study suggest a slightly increased risk of glioma after use of penicillins, but not of other antibiotics. However, an OR of 1.1 derived from observational data may be explained by residual bias or confounding and cannot be considered to be a clinically important finding. Furthermore, underlying infections could also explain the observed associations. To address this, we included various infectious diseases in our multivariable model. This did not alter the risk estimates, but we cannot rule out residual confounding.

There was no dose–response found in this analysis. The effect of antibiotics on the risk of glioma did not change with increased number of prescriptions. However, as antibiotic drug use leads to long‐lasting alterations of the intestinal microbiota,^25^, ^34^, ^35^ a single prescription could in theory result in long‐term perturbations of serum levels of SCFAs, tryptophane metabolites, and secondary bile acids. We did not find evidence for this in this study.

Interestingly, we observed an inverse relation between use of polyenes and risk of glioma. Polyenes are broad‐spectrum antifungal drugs acting by formation of ion‐channel like complexes in interaction with ergosterol.^45^ In spite of its high toxicity, amphotericin B, and nystatin are effective systemically acting polyenes still in clinical use for treatment of invasive fungal infections.^45^ We found no previous epidemiologic study that investigated associations between the use of polyenes and glioma incidence. Experimental data show that amphotericin B is able to inhibit sphere formation in brain tumor‐initiating cells in vitro by activation of microglia and macrophages.^46^ Mice with glioma that receive amphotericin B show longer survival periods.^46^ Another study with rat glioma cell lines also suggests anti‐tumor efficacy of Amphotericin B.^47^ We were not able to document a dose–response relation in our study, but only 12 glioma cases received five or more prescriptions compared to 115 cases that received one prescription. Due to their high toxicity, polyenes are usually restricted to severely ill patients with no other therapeutic alternatives.^45^ This might lead to lower detection rates of subsequent glioma in these patients whose prognosis is limited by severe infection. This is supported by our observation that cases with an increasingly long period since their first prescription of polyenes showed a reduced risk of glioma.

Epilepsy, DVT, and use of opioids were associated with a higher risk of glioma. These associations could be explained by reverse causation in patients with undiagnosed glioma, because these conditions were recorded before the index date. In order to reduce the possibility of reverse causation and to account for the lag time between tumor development and detection, the index date was set 1 year before diagnosis of glioma.

Cases and controls with Human Immuno deficiency Virus [HIV] infection were excluded from the study and we could not identify any cases with HIV antiviral prescriptions. However, we identified four controls (0.009%) that received HIV antivirals. Other indications for these prescriptions like post‐exposition prophylaxis are possible. However, we cannot fully rule out missing diagnosis coding.

This study has some limitations. Lack of hospital data is the main limitation. Specifically, we might have missed information on severely ill patients with repeated antimicrobial drug administration over a long period. Similarly, we did not have information on the use of antibiotics in hospital and may have missed the use of intravenously administered antibiotics such as glycopeptides, which are administered predominantly in inpatient care. However, in 2020, around 72.7% of all antibiotics in England were prescribed by general practitioners and only 12.8% by hospital physicians.^48^ Antibiotics lead to alterations of the intestinal microbiota. However, alterations of the intestinal microbiota could also occur after other potentially confounding environmental changes such as the intake of probiotics as a dietary measure. This represents a limitation of our study, because, unlike antibiotics, these products are usually available over the counter and therefore not recorded by general practitioners. Another limitation of our study is possible non‐compliance with the prescribed drugs. Self‐reported non‐adherence was described as being around 10%–11% in two European countries.^49^ However, non‐compliance includes missing single doses or leftover doses in most cases. It is unlikely that not even a single dose of a prescription is taken. In addition, all data that were investigated in this study were recorded by general practitioners. Thus, missing or delayed documentation of prescriptions or misclassification of diagnoses is possible. Nonetheless, this is unlikely to play an important role, as the validity of the CPRD GOLD database has been demonstrated multiple times,^29^ and general practitioners often record diagnoses supplied by oncologists. Another potential limitation is that many years of life may not be covered by the database as cases and controls have a mean age of 54 years and a mean history of 11.8 years prior to the index date. This means that the exposure assessment in this study is based on a considerably long observation period, but does not reflect the life‐time prevalence of antimicrobial use in cases and controls. We only considered patients with at least 3 years of active history in the database and we showed detailed analyses for the association of antimicrobial drug use with glioma incidence stratified by time after diagnosis covering over 15 years. Furthermore, if misclassification occurred, it was most likely non‐differential, which would have biased the potential effect toward the null. Thus, an increased risk of glioma may have been weakened to some degree by misclassification. Furthermore, adjustment for socioeconomic status was not possible because such information is not regularly recorded in the CPRD. In order to account for this, we matched controls to cases based on general practice, because persons of the same geographic area tend to be of a comparable socioeconomic status. The association between ever‐use of antibiotics and glioma risk was most pronounced in patients older than 60 years of age. The incidence of glioma is higher in older patients^2^ and older patients are more likely to have ever received antibiotics, leading to possible bias. However, our statistical test for interaction by age remained non‐significant.

Our study has several strengths. To date, no previous study has provided specific insights on the association between antimicrobial drug use and incidence of glioma. The CPRD includes data on more than 11 million patients with acceptable quality for research purposes.^28^ We were able to perform a large case–control study with 4423 glioma cases. Another major strength of our study is that, in contrast to many other case–control studies, recall bias caused by self‐report plays only a minor role, since the data in the CPRD are prospectively collected by general practitioners.^28^

In conclusion, we investigated associations between antimicrobial drugs and the risk of subsequent glioma in a large population‐based case–control study with 4423 glioma cases and 44,230 controls. We did not find evidence for an increased risk of glioma in ever‐use of antibiotics as a group or in subgroup analyses. We observed an inverse association between use of polyenes and incidence of glioma. Further research is necessary to independently confirm these results and to clarify possible underlying mechanisms that could be harnessed for therapeutic interventions.

## AUTHOR CONTRIBUTIONS

**Christoph R. Meier:** Data curation (equal); project administration (equal). **Claudia Becker:** Data curation (equal); project administration (equal). **Corinna Seliger:** Conceptualization (equal); methodology (equal); supervision (equal). **Michael Leitzmann:** Conceptualization (equal); methodology (equal); resources (lead). **Peter Hau:** Conceptualization (equal); supervision (equal). **Ralf Linker:** Conceptualization (equal). **Susan Jick:** Data curation (equal); project administration (equal). **Tareq Marius Haedenkamp:** Conceptualization (equal); formal analysis (lead); methodology (equal); writing – original draft (lead).

## ETHICS APPROVAL

This study was approved by the Independent Scientific Advisory Committee for Medicines and Healthcare products Regulatory Agency database research (protocol no: 19_189). Informed consent was not required as the Clinical Practice Research Datalink (CPRD) provides anonymized data from medical records. This study is based in part on data from the Clinical Practice Research Datalink obtained under license from the UK Medicines and Healthcare products Regulatory Agency. The data is provided by patients and collected by the NHS as part of their care and support. The interpretation and conclusions contained in this study are those of the author/s alone.

## Supporting information


Table S1

Table S2

Table S3

## Data Availability

The data that support the findings of this study are available from the Clinical Practice Research Datalink (CPRD). Restrictions apply to the availability of these data, which were used under license for this study. Data are available from the authors with the permission of the CPRD.
