# Supplementary material for: Antimicrobial drug use and the risk of glioma: A case–control study
Source: Cancer Med. 2022 Sep 6;12(3):3684–95. doi: 10.1002/cam4.5222 (PMC9939229; doi:10.1002/cam4.5222)
Supplement: Supplementary file 1 — Table S1 [file CAM4-12-3684-s003.docx]

# Supplementary Table 1: Read codes for glioma

BBb..00 Gliomas

BBb0.00 Glioma; malignant

BBb0.11 Glioma NOS

BBb0.12 Gliosarcoma

BBb1.00 Gliomatosis cerebri

BBb2.00 Mixed glioma

BBb2.11 Mixed glioma

BBb3.00 Subependymal glioma

BBb3.11 Subependymal astrocytoma NOS

BBb3.12 Subependymal astrocytoma NOS

BBb3.13 Subependymoma

BBb4.00 Subependymal giant cell astrocytoma

BBb7.00 Ependymoma NOS

BBb8.00 Ependymoma, anaplastic type

BBb8.11 Ependymoblastoma

BBb9.00 Papillary ependymoma

BBbA.00 Myxopapillary ependymoma

BBbB.00 Astrocytoma NOS

BBbB.11 Astrocytic glioma

BBbC.00 Astrocytoma, anaplastic type

BBbD.00 Protoplasmic astrocytoma

BBbE.00 Gemistocytic astrocytoma

BBbE.11 Gemistocytoma

BBbF.00 Fibrillary astrocytoma

BBbG.00 Pilocytic astrocytoma

BBbG.11 Juvenile astrocytoma

BBbG.12 Piloid astrocytoma

BBbK.00 Astroblastoma

BBbL.00 Glioblastoma NOS

BBbL.11 Glioblastoma multiforme

BBbM.00 Giant cell glioblastoma

BBbN.00 Glioblastoma with sarcomatous component

BBbQ.00 Oligodendroglioma NOS

BBbR.00 Oligodendroglioma, anaplastic type

BBbS.00 Oligodendroblastoma

BBbz.00 Glioma NOS

BBbZ.00 Pleomorphic xanthoastrocytoma
