# Supplementary material for: Antimicrobial drug use and the risk of glioma: A case–control study
Source: Cancer Med. 2022 Sep 6;12(3):3684–95. doi: 10.1002/cam4.5222 (PMC9939229; doi:10.1002/cam4.5222)
Supplement: Supplementary file 2 — Table S2 [file CAM4-12-3684-s002.docx]

| **Supplementary Table 2: Number of prescriptions of antimicrobial drugs and risk of glioma** | | | | | | | |
| --- | --- | --- | --- | --- | --- | --- | --- |
|  |  | |  |  |  |  |  |
|  |  | |  | cases (n=4423) | controls (n=44230) | Adjusted OR (95% CI) | |
|  |  | |  | number (%) | number (%) |  |  |
| **Number of prescriptions** |  | |  |  |  |  |  |
|  |  | |  |  |  |  |  |
| Antibiotics/Antibacterials |  | |  |  |  |  |  |
|  | 0 | |  | 932 (21.1) | 10018 (22.7) | 1.00 (reference) |  |
|  | 1 | |  | 629 (14.2) | 6080 (13.8) | **1.13 (1.01-1.26)** |  |
|  | 2-4 | |  | 1215 (27.5) | 11788 (26.7) | **1.14 (1.03-1.27)** |  |
|  | >=5 | |  | 1647 (37.2) | 16344 (37.0) | **1.12 (1.00-1.25)** |  |
|  | p value for trend | | |  |  |  | 0.422 |
|  |  | |  |  |  |  |  |
| Antivirals |  | |  |  |  |  |  |
|  | 0 | |  | 4231 (95.7) | 42260 (95.6) | 1.00 (reference) |  |
|  | 1 | |  | 156 (3.5) | 1674 (3.8) | 0.93 (0.78-1.10) |  |
|  | 2-4 | |  | 29 (0.7) | 230 (0.5) | 1.21 (0.82-1.79) |  |
|  | >=5 | |  | 7 (0.2) | 66 (0.2) | 1.00 (0.46-2.20) |  |
|  | p value for trend | | |  |  |  | 0.952 |
|  |  | |  |  |  |  |  |
| Antifungals |  | |  |  |  |  |  |
|  | 0 | |  | 3456 (78.1) | 34679 (78.4) | 1.00 (reference) |  |
|  | 1 | |  | 460 (10.4) | 4440 (10.0) | 1.03 (0.92-1.15) |  |
|  | 2-4 | |  | 352 (8.0) | 3459 (7.8) | 1.00 (0.88-1.14) |  |
|  | >=5 | |  | 155 (3.5) | 1652 (3.7) | 0.93 (0.77-1.11) |  |
|  | p value for trend | | |  |  |  | 0.457 |
|  |  | |  |  |  |  |  |
| Antiprotozoals |  | |  |  |  |  |  |
|  | 0 | |  | 4104 (92.8) | 41111 (93.0) | 1.00 (reference) |  |
|  | 1 | |  | 160 (3.6) | 1465 (3.3) | 1.08 (0.91-1.28) |  |
|  | 2-4 | |  | 73 (1.7) | 793 (1.8) | 0.92 (0.72-1.17) |  |
|  | >=5 | |  | 86 (1.9) | 861 (2.0) | 1.00 (0.79-1.26) |  |
|  | p value for trend | | |  |  |  | 0.978 |
|  |  | |  |  |  |  |  |
| **Antibiotics by mechanism** | | |  |  |  |  |  |
|  | Bactericidal | |  |  |  |  |  |
|  | 0 | |  | 1106 (25.0) | 11827 (26.7) | 1.00 (reference) |  |
|  | 1 | |  | 705 (15.9) | 6851 (15.5) | **1.12 (1.01-1.24)** |  |
|  | 2-4 | |  | 1255 (28.4) | 12257 (27.7) | **1.13 (1.03-1.24)** |  |
|  | >=5 | |  | 1357 (30.7) | 13295 (30.1) | **1.13 (1.02-1.27)** |  |
|  | p value for trend | | |  |  |  | 0.145 |
|  |  | |  |  |  |  |  |
|  | Bacteriostatic | | |  |  |  |  |
|  | 0 | |  | 3086 (69.8) | 30814 (69.7) | 1.00 (reference) |  |
|  | 1 | |  | 662 (15.0) | 6785 (15.3) | 0.97 (0.88-1.06) |  |
|  | 2-4 | |  | 490 (11.1) | 4820 (10.9) | 1.01 (0.91-1.13) |  |
|  | >=5 | |  | 185 (4.2) | 1811 (4.1) | 1.01 (0.86-1.19) |  |
|  | p value for trend | | |  |  |  | 0.907 |
|  |  | |  |  |  |  |  |
|  | Cell wall inhibitors | | | |  |  |  |
|  | 0 | |  | 1229 (27.8) | 12979 (29.3) | 1.00 (reference) |  |
|  | 1 | |  | 763 (17.3) | 7406 (16.7) | **1.10 (1.00-1.22)** |  |
|  | 2-4 | |  | 1230 (27.8) | 12188 (27.6) | **1.10 (1.00-1.20)** |  |
|  | >=5 | |  | 1201 (27.2) | 11657 (26.4) | **1.12 (1.01-1.25)** |  |
|  | p value for trend | | |  |  |  | 0.116 |
|  |  | |  |  |  |  |  |
|  | Inhibitors of protein synthesis | | | |  |  |  |
|  | 0 | |  | 2686 (60.7) | 26720 (60.4) | 1.00 (reference) |  |
|  | 1 | |  | 735 (16.6) | 7408 (16.8) | 0.98 (0.90-1.07) |  |
|  | 2-4 | |  | 645 (14.6) | 6608 (14.9) | 0.96 (0.87-1.06) |  |
|  | >=5 | |  | 357 (8.1) | 3494 (7.9) | 1.00 (0.89-1.14) |  |
|  | p value for trend | | |  |  |  | 0.974 |
|  |  | |  |  |  |  |  |
|  | Inhibitors of DNA/RNA synthesis | | | |  |  |  |
|  | 0 | |  | 3401 (76.9) | 33846 (76.5) | 1.00 (reference) |  |
|  | 1 | |  | 548 (12.4) | 5531 (12.5) | 0.97 (0.88-1.08) |  |
|  | 2-4 | |  | 367 (8.3) | 3645 (8.2) | 0.98 (0.87-1.11) |  |
|  | >=5 | |  | 107 (2.4) | 1208 (2.7) | 0.86 (0.70-1.06) |  |
|  | p value for trend | | |  |  |  |  |
|  |  | |  |  |  |  |  |
|  | Inhibitors of folic acid synthesis | | | |  |  |  |
|  | 0 | |  | 4283 (96.8) | 42996 (97.2) | 1.00 (reference) |  |
|  | 1 | |  | 94 (2.1) | 888 (2.0) | 1.09 (0.87-1.36) |  |
|  | 2-4 | |  | 38 (0.9) | 292 (0.7) | 1.34 (0.94-1.91) |  |
|  | >=5 | |  | 8 (0.2) | 54 (0.1) | 1.46 (0.69-3.10) |  |
|  | p value for trend | | |  |  |  | 0.078 |
|  |  | |  |  |  |  |  |
| **Specific antibiotics** |  | |  |  |  |  |  |
|  |  | |  |  |  |  |  |
|  | Penicillins | |  |  |  |  |  |
|  | 0 | |  | 1365 (30.9) | 14359 (32.5) | 1.00 (reference) |  |
|  | 1 | |  | 785 (17.8) | 7599 (17.2) | **1.10 (1.00-1.21)** |  |
|  | 2-4 | |  | 1202 (27.2) | 12002 (27.1) | 1.08 (0.99-1.18) |  |
|  | >=5 | |  | 1071 (24.2) | 10270 (23.2) | **1.13 (1.02-1.26)** |  |
|  | p value for trend | | |  |  |  | 0.058 |
|  |  | |  |  |  |  |  |
|  | Cephalosporins and Beta lactams | | | |  |  |  |
|  | 0 | |  | 3557 (80.4) | 35515 (80.3) | 1.00 (reference) |  |
|  | 1 | |  | 481 (10.9) | 4796 (10.8) | 0.98 (0.88-1.09) |  |
|  | 2-4 | |  | 291 (6.6) | 2886 (6.5) | 0.98 (0.86-1.12) |  |
|  | >=5 | |  | 94 (2.1) | 1033 (2.3) | 0.88 (0.70-1.10) |  |
|  | p value for trend | | |  |  |  | 0.261 |
|  |  | |  |  |  |  |  |
|  | Macrolides | |  |  |  |  |  |
|  | 0 | |  | 3162 (71.5) | 31405 (71.0) | 1.00 (reference) |  |
|  | 1 | |  | 636 (14.4) | 6595 (14.9) | 0.95 (0.87-1.04) |  |
|  | 2-4 | |  | 458 (10.4) | 4588 (10.4) | 0.98 (0.88-1.10) |  |
|  | >=5 | |  | 167 (3.8) | 1642 (3.7) | 1.00 (0.84-1.18) |  |
|  | p value for trend | | |  |  |  | 0.857 |
|  |  | |  |  |  |  |  |
|  | Tetracyclines | | |  |  |  |  |
|  | 0 | |  | 3500 (79.1) | 34870 (78.8) | 1.00 (reference) |  |
|  | 1 | |  | 472 (10.7) | 4774 (10.8) | 0.99 (0.89-1.10) |  |
|  | 2-4 | |  | 300 (6.8) | 3131 (7.1) | 0.94 (0.83-1.08) |  |
|  | >=5 | |  | 151 (3.4) | 1455 (3.3) | 1.02 (0.86-1.22) |  |
|  | p value for trend | | |  |  |  | 0.934 |
|  |  | |  |  |  |  |  |
|  | Nitrofurantoin | | |  |  |  |  |
|  | 0 | |  | 4245 (96.0) | 42499 (96.1) | 1.00 (reference) |  |
|  | 1 | |  | 121 (2.7) | 1051 (2.4) | 1.12 (0.92-1.37) |  |
|  | 2-4 | |  | 44 (1.0) | 486 (1.1) | 0.89 (0.65-1.23) |  |
|  | >=5 | |  | 13 (0.3) | 194 (0.4) | 0.65 (0.37-1.15) |  |
|  | p value for trend | | |  |  |  | 0.178 |
|  |  | |  |  |  |  |  |
|  | Nitroimidazole derivates | | | |  |  |  |
|  | 0 | |  | 3700 (83.7) | 36900 (83.4) | 1.00 (reference) |  |
|  | 1 | |  | 438 (9.9) | 4418 (10.0) | 0.98 (0.88-1.09) |  |
|  | 2-4 | |  | 233 (5.3) | 2373 (5.4) | 0.97 (0.84-1.12) |  |
|  | >=5 | |  | 52 (1.2) | 539 (1.2) | 0.94 (0.70-1.27) |  |
|  | p value for trend | | |  |  |  | 0.552 |
|  |  | |  |  |  |  |  |
|  | Quinolones | |  |  |  |  |  |
|  | 0 | |  | 3928 (88.8) | 39300 (88.9) | 1.00 (reference) |  |
|  | 1 | |  | 314 (7.1) | 3079 (7.0) | 1.01 (0.89-1.14) |  |
|  | 2-4 | |  | 135 (3.1) | 1408 (3.2) | 0.94 (0.78-1.13) |  |
|  | >=5 | |  | 46 (1.0) | 443 (1.0) | 1.05 (0.77-1.44) |  |
|  | p value for trend | | |  |  |  | 0.947 |
|  |  | |  |  |  |  |  |
| **Specific antifungals** |  | |  |  |  |  |  |
|  | Triazoles | |  |  |  |  |  |
|  | 0 | |  | 3704 (83.7) | 37056 (83.8) | 1.00 (reference) |  |
|  | 1 | |  | 383 (8.7) | 3737 (8.5) | 1.02 (0.90-1.14) |  |
|  | 2-4 | |  | 241 (5.5) | 2419 (5.5) | 0.97 (0.83-1.13) |  |
|  | >=5 | |  | 95 (2.2) | 1018 (2.3) | 0.89 (0.70-1.13) |  |
|  | p value for trend | | |  |  |  | 0.348 |
|  |  | |  |  |  |  |  |
|  | Polyenes | |  |  |  |  |  |
|  | 0 | |  | 4254 (96.2) | 42213 (95.4) | 1.00 (reference) |  |
|  | 1 | |  | 115 (2.6) | 1405 (3.2) | 0.79 (0.65-0.97) |  |
|  | 2-4 | |  | 42 (1.0) | 504 (1.1) | 0.81 (0.59-1.13) |  |
|  | >=5 | |  | 12 (0.3) | 108 (0.2) | 1.09 (0.59-2.00) |  |
|  | p value for trend | | |  |  |  | 0.277 |
|  |  | |  |  |  |  |  |
|  | Other antifungals | | |  |  |  |  |
|  | 0 | |  | 4231 (95.7) | 42443 (96.0) | 1.00 (reference) |  |
|  | 1 | |  | 69 (1.6) | 624 (1.4) | 1.10 (0.85-1.41) |  |
|  | 2-4 | |  | 82 (1.9) | 784 (1.8) | 1.04 (0.82-1.31) |  |
|  | >=5 | |  | 41 (0.9) | 379 (0.9) | 1.10 (0.80-1.53) |  |
|  | p value for trend | | |  |  |  | 0.461 |
|  |  | |  |  |  |  |  |
| **Topical antimicrobial drugs** | | |  |  |  |  |  |
|  | Topical antibiotics | | | |  |  |  |
|  | 0 | |  | 1889 (42.7) | 19648 (44.4) | 1.00 (reference) |  |
|  | 1 | |  | 911 (20.6) | 8273 (18.7) | **1.15 (1.05-1.25)** |  |
|  | 2-4 | |  | 924 (20.9) | 9529 (21.5) | 1.00 (0.92-1.09) |  |
|  | >=5 | |  | 699 (15.8) | 6780 (15.3) | 1.06 (0.96-1.17) |  |
|  | p value for trend | | |  |  |  | 0.698 |
|  |  | |  |  |  |  |  |
|  | Topical antifungals | | | |  |  |  |
|  | 0 | |  | 2571 (58.1) | 26034 (58.9) | 1.00 (reference) |  |
|  | 1 | |  | 747 (16.9) | 7231 (16.4) | 1.04 (0.95-1.13) |  |
|  | 2-4 | |  | 681 (15.4) | 6781 (15.3) | 1.01 (0.92-1.11) |  |
|  | >=5 | |  | 424 (9.6) | 4184 (9.5) | 1.00 (0.89-1.13) |  |
|  | p value for trend | | |  |  |  | 0.959 |
|  |  | |  |  |  |  |  |
|  | Topical antivirals | | |  |  |  |  |
|  | 0 | |  | 4218 (95.4) | 42499 (96.1) | 1.00 (reference) |  |
|  | 1 | |  | 126 (2.9) | 1104 (2.5) | 1.14 (0.94-1.38) |  |
|  | 2-4 | |  | 57 (1.3) | 427 (1.0) | 1.32 (1.00-1.76) |  |
|  | >=5 | |  | 22 (0.5) | 200 (0.5) | 1.09 (0.70-1.71) |  |
|  | p value for trend | | |  |  |  | 0.199 |
|  |  | | |  |  |  |  |
|  |  | | |  |  |  |  |
| adjusted for: | BMI, smoking, diabetes, congestive heart failure, myocardial infarction, deep | | | | | | |
|  | vein thrombosis, epilepsy, renal disease, opioid use, and infectious diseases | | | | | | |
|  |  | | | | | | |
|  | We performed Bonferroni correction for multiple testing of p value thresholds. Significant results after Bonferroni correction are indicated by an asterisk (*). | | | | | | |
|  |  |  |  |  |  |  |  |
|  | |  | | | | | |
