# Supplementary material for: Antimicrobial drug use and the risk of glioma: A case–control study
Source: Cancer Med. 2022 Sep 6;12(3):3684–95. doi: 10.1002/cam4.5222 (PMC9939229; doi:10.1002/cam4.5222)
Supplement: Supplementary file 3 — Table S3 [file CAM4-12-3684-s001.docx]

| **Supplementary Table 3: Time since first prescription of antimicrobial drugs and risk of glioma** | | | | | | |
| --- | --- | --- | --- | --- | --- | --- |
|  |  |  |  |  |  |  |
|  |  |  | cases (n=4423) | controls (n=44230) | Adjusted OR (95% CI) | |
|  | |  | number (%) | number (%) |  |  |
|  | |  |  |  |  |  |
| **Time since first prescription [years]** | | | |  |  |  |
| Antibiotics/Antibacterials | |  |  |  |  |  |
|  | no prescription |  | 932 (21.1) | 10018 (22.7) | 1.00 (reference) |  |
|  | 1-5 |  | 881 (19.9) | 8746 (19.8) | 1.10 (0.99-1.22) |  |
|  | 6-10 |  | 1151 (26.0) | 10879 (24.6) | **1.20 (1.07-1.33)** |  |
|  | 11-15 |  | 769 (17.4) | 7682 (17.4) | 1.12 (0.98-1.28) |  |
|  | >15 |  | 690 (15.6) | 6905 (15.6) | 1.11 (0.94-1.31) |  |
|  | p value for trend | |  |  |  | 0.095 |
|  |  |  |  |  |  |  |
| Antivirals |  |  |  |  |  |  |
|  | no prescription |  | 4231 (95.7) | 42260 (95.6) | 1.00 (reference) |  |
|  | 1-5 |  | 94 (2.1) | 1005 (2.3) | 0.93 (0.75-1.15) |  |
|  | 6-10 |  | 59 (1.3) | 586 (1.3) | 0.99 (0.76-1.30) |  |
|  | 11-15 |  | 31 (0.7) | 270 (0.6) | 1.11 (0.76-1.62) |  |
|  | >15 |  | 8 (0.2) | 109 (0.3) | 0.72 (0.35-1.50) |  |
|  | p value for trend | |  |  |  | 0.752 |
|  |  |  |  |  |  |  |
| Antifungals |  |  |  |  |  |  |
|  | no prescription |  | 3456 (78.1) | 34679 (78.4) | 1.00 (reference) |  |
|  | 1-5 |  | 382 (8.6) | 3540 (8.0) | 1.06 (0.95-1.20) |  |
|  | 6-10 |  | 284 (6.4) | 2901 (6.6) | 0.97 (0.85-1.12) |  |
|  | 11-15 |  | 165 (3.7) | 1765 (4.0) | 0.93 (0.78-1.11) |  |
|  | >15 |  | 136 (3.1) | 1345 (3.0) | 1.00 (0.81-1.24) |  |
|  | p value for trend | |  |  |  | 0.622 |
|  |  |  |  |  |  |  |
| Antiprotozoals |  |  |  |  |  |  |
|  | no prescription |  | 4104 (92.8) | 41111 (93.0) | 1.00 (reference) |  |
|  | 1-5 |  | 146 (3.3) | 1360 (3.1) | 1.07 (0.90-1.28) |  |
|  | 6-10 |  | 99 (2.2) | 983 (2.2) | 1.01 (0.81-1.24) |  |
|  | 11-15 |  | 50 (1.1) | 503 (1.1) | 0.97 (0.72-1.31) |  |
|  | >15 |  | 24 (0.5) | 273 (0.6) | 0.87 (0.57-1.33) |  |
|  | p value for trend | |  |  |  | 0.717 |
|  |  |  |  |  |  |  |
| **Antibiotics by mechanism** |  |  |  |  |  |  |
|  | Bactericidal |  |  |  |  |  |
|  | no prescription |  | 1106 (25.0) | 11827 (26.7) | 1.00 (reference) |  |
|  | 1-5 |  | 909 (20.6) | 8914 (20.2) | **1.10 (1.00-1.22)** |  |
|  | 6-10 |  | 1086 (24.6) | 10347 (23.4) | **1.17 (1.05-1.29)** |  |
|  | 11-15 |  | 699 (15.8) | 7053 (16.0) | 1.10 (0.97-1.25) |  |
|  | >15 |  | 623 (14.1) | 6089 (13.8) | 1.16 (0.99-1.36) |  |
|  | p value for trend | |  |  |  | **0.045** |
|  |  |  |  |  |  |  |
|  | Bacteriostatic |  |  |  |  |  |
|  | no prescription |  | 3086 (69.8) | 30814 (69.7) | 1.00 (reference) |  |
|  | 1-5 |  | 456 (10.3) | 4552 (10.3) | 0.99 (0.89-1.11) |  |
|  | 6-10 |  | 432 (9.8) | 4123 (9.3) | 1.04 (0.93-1.17) |  |
|  | 11-15 |  | 256 (5.8) | 2650 (6.0) | 0.95 (0.83-1.10) |  |
|  | >15 |  | 193 (4.4) | 2091 (4.7) | 0.89 (0.74-1.06) |  |
|  | p value for trend | |  |  |  | 0.318 |
|  |  |  |  |  |  |  |
|  | Cell wall inhibitors | | |  |  |  |
|  | no prescription |  | 1229 (27.8) | 12979 (29.3) | 1.00 (reference) |  |
|  | 1-5 |  | 878 (19.9) | 8623 (19.5) | 1.08 (0.98-1.19) |  |
|  | 6-10 |  | 1049 (23.7) | 9948 (22.5) | **1.15 (1.04-1.27)** |  |
|  | 11-15 |  | 668 (15.1) | 6786 (15.3) | 1.06 (0.93-1.20) |  |
|  | >15 |  | 599 (13.5) | 5894 (13.3) | 1.11 (0.95-1.29) |  |
|  | p value for trend | |  |  |  | 0.128 |
|  |  |  |  |  |  |  |
|  | Inhibitors of protein synthesis | | |  |  |  |
|  | no prescription |  | 2686 (60.7) | 26721 (60.4) | 1.00 (reference) |  |
|  | 1-5 |  | 553 (12.5) | 5660 (12.8) | 0.97 (0.88-1.07) |  |
|  | 6-10 |  | 563 (12.7) | 5305 (12.0) | 1.05 (0.95-1.17) |  |
|  | 11-15 |  | 355 (8.0) | 3637 (8.2) | 0.95 (0.84-1.08) |  |
|  | >15 |  | 266 (6.0) | 2907 (6.6) | 0.86 (0.74-1.01) |  |
|  | p value for trend | |  |  |  | 0.194 |
|  |  |  |  |  |  |  |
|  | Inhibitors of DNA/RNA synthesis | | |  |  |  |
|  | no prescription |  | 3401 (76.9) | 33846 (76.5) | 1.00 (reference) |  |
|  | 1-5 |  | 435 (9.8) | 4487 (10.1) | 0.95 (0.86-1.06) |  |
|  | 6-10 |  | 312 (7.1) | 3173 (7.2) | 0.97 (0.85-1.10) |  |
|  | 11-15 |  | 184 (4.2) | 1723 (3.9) | 1.05 (0.89-1.24) |  |
|  | >15 |  | 91 (2.1) | 1001 (2.3) | 0.86 (0.68-1.09) |  |
|  | p value for trend | |  |  |  | 0.488 |
|  |  |  |  |  |  |  |
|  | Inhibitors of folic acid synthesis | | |  |  |  |
|  | no prescription |  | 4283 (96.8) | 42996 (97.2) | 1.00 (reference) |  |
|  | 1-5 |  | 11 (0.3) | 129 (0.3) | 0.82 (0.43-1.56) |  |
|  | 6-10 |  | 45 (1.0) | 327 (0.7) | **1.45 (1.03-2.02)** |  |
|  | 11-15 |  | 38 (0.9) | 325 (0.7) | 1.22 (0.85-1.75) |  |
|  | >15 |  | 46 (1.0) | 453 (1.0) | 1.03 (0.74-1.43) |  |
|  | p value for trend | |  |  |  | 0.248 |
|  |  |  |  |  |  |  |
| **Specific antibiotics** |  |  |  |  |  |  |
|  | Penicillins |  |  |  |  |  |
|  | no prescription |  | 1365 (30.9) | 14359 (32.5) | 1.00 (reference) |  |
|  | 1-5 |  | 863 (19.5) | 8428 (19.1) | 1.08 (0.98-1.19) |  |
|  | 6-10 |  | 995 (22.5) | 9485 (21.4) | **1.13 (1.03-1.25)** |  |
|  | 11-15 |  | 638 (14.4) | 6426 (14.5) | 1.06 (0.94-1.20) |  |
|  | >15 |  | 562 (12.7) | 5532 (12.5) | 1.10 (0.94-1.20) |  |
|  | p value for trend | |  |  |  | 0.129 |
|  |  |  |  |  |  |  |
|  | Cephalosporins and Beta lactams | | |  |  |  |
|  | no prescription |  | 3557 (80.4) | 35515 (80.3) | 1.00 (reference) |  |
|  | 1-5 |  | 291 (6.6) | 2867 (6.5) | 1.00 (0.87-1.14) |  |
|  | 6-10 |  | 286 (6.5) | 2758 (6.2) | 1.01 (0.88-1.16) |  |
|  | 11-15 |  | 161 (3.6) | 1785 (4.0) | 0.88 (0.74-1.05) |  |
|  | >15 |  | 128 (2.9) | 1305 (3.0) | 0.94 (0.76-1.16) |  |
|  | p value for trend | |  |  |  | 0.303 |
|  |  |  |  |  |  |  |
|  | Macrolides |  |  |  |  |  |
|  | no prescription |  | 3162 (71.5) | 31405 (71.0) | 1.00 (reference) |  |
|  | 1-5 |  | 457 (10.3) | 4597 (10.4) | 0.98 (0.88-1.09) |  |
|  | 6-10 |  | 408 (9.2) | 3954 (8.9) | 1.02 (0.91-1.14) |  |
|  | 11-15 |  | 231 (5.2) | 2476 (5.6) | 0.91 (0.78-1.06) |  |
|  | >15 |  | 165 (3.7) | 1798 (4.1) | 0.87 (0.72-1.05) |  |
|  | p value for trend | |  |  |  | 0.138 |
|  |  |  |  |  |  |  |
|  | Sulphonamides and trimethoprim | | |  |  |  |
|  | no prescription |  | 4283 (96.8) | 42996 (97.2) | 1.00 (reference) |  |
|  | 1-5 |  | 11 (0.3) | 129 (0.3) | 0.82 (0.43-1.56) |  |
|  | 6-10 |  | 45 (1.0) | 327 (0.7) | **1.45 (1.03-2.02)** |  |
|  | 11-15 |  | 38 (0.9) | 325 (0.7) | 1.22 (0.85-1.75) |  |
|  | >15 |  | 46 (1.0) | 453 (1.0) | 1.03 (0.74-1.43) |  |
|  | p value for trend | |  |  |  | 0.248 |
|  |  |  |  |  |  |  |
|  | Tetracyclines |  |  |  |  |  |
|  | no prescription |  | 3500 (79.1) | 34871 (78.8) | 1.00 (reference) |  |
|  | 1-5 |  | 305 (6.9) | 3155 (7.1) | 0.97 (0.85-1.10) |  |
|  | 6-10 |  | 290 (6.6) | 2734 (6.2) | 1.06 (0.93-1.21) |  |
|  | 11-15 |  | 191 (4.3) | 1881 (4.3) | 0.99 (0.84-1.17) |  |
|  | >15 |  | 137 (3.1) | 1589 (3.6) | 0.83 (0.68-1.01) |  |
|  | p value for trend |  |  |  |  | 0.267 |
|  |  |  |  |  |  |  |
|  | Nitrofurantoin |  |  |  |  |  |
|  | no prescription |  | 4245 (96.0) | 42499 (96.1) | 1.00 (reference) |  |
|  | 1-5 |  | 122 (2.8) | 1062 (2.4) | 1.14 (0.93-1.39) |  |
|  | 6-10 |  | 36 (0.8) | 428 (1.0) | 0.81 (0.57-1.15) |  |
|  | 11-15 |  | 15 (0.3) | 158 (0.4) | 0.90 (0.52-1.55) |  |
|  | >15 |  | 5 (0.1) | 83 (0.2) | 0.58 (0.23-1.45) |  |
|  | p value for trend |  |  |  |  | 0.203 |
|  |  |  |  |  |  |  |
|  | Nitroimidazole derivates | | |  |  |  |
|  | no prescription |  | 3700 (83.7) | 36900 (83.4) | 1.00 (reference) |  |
|  | 1-5 |  | 332 (7.5) | 3411 (7.7) | 0.97 (0.85-1.09) |  |
|  | 6-10 |  | 215 (4.9) | 2159 (4.9) | 0.99 (0.85-1.14) |  |
|  | 11-15 |  | 121 (2.7) | 1109 (2.5) | 1.08 (0.88-1.32) |  |
|  | >15 |  | 55 (1.2) | 651 (1.5) | 0.80 (0.59-1.08) |  |
|  | p value for trend | | |  |  | 0.514 |
|  |  |  |  |  |  |  |
|  | Quinolones |  |  |  |  |  |
|  | no prescription |  | 3928 (88.8) | 39300 (88.9) | 1.00 (reference) |  |
|  | 1-5 |  | 204 (4.6) | 2167 (4.9) | 0.93 (0.80-1.09) |  |
|  | 6-10 |  | 158 (3.6) | 1490 (3.4) | 1.05 (0.88-1.24) |  |
|  | 11-15 |  | 91 (2.1) | 819 (1.9) | 1.11 (0.89-1.39) |  |
|  | >15 |  | 42 (1.0) | 454 (1.0) | 0.91 (0.65-1.26) |  |
|  | p value for trend | |  |  |  | 0.799 |
|  |  |  |  |  |  |  |
|  |  |  |  |  |  |  |
| **Specific antifungals** | |  |  |  |  |  |
|  | Triazoles |  |  |  |  |  |
|  | no prescription |  | 3704 (83.7) | 37056 (83.8) | 1.00 (reference) |  |
|  | 1-5 |  | 273 (6.2) | 2579 (5.8) | 1.04 (0.91-1.19) |  |
|  | 6-10 |  | 203 (4.6) | 2164 (4.9) | 0.93 (0.80-1.09) |  |
|  | 11-15 |  | 131 (3.0) | 1329 (3.0) | 0.98 (0.80-1.20) |  |
|  | >15 |  | 112 (2.5) | 1102 (2.5) | 1.00 (0.80-1.26) |  |
|  | p value for trend | |  |  |  | 0.705 |
|  |  |  |  |  |  |  |
|  | Polyenes |  |  |  |  |  |
|  | no prescription |  | 4254 (96.2) | 42213 (95.4) | 1.00 (reference) |  |
|  | 1-5 |  | 82 (1.9) | 899 (2.0) | 0.88 (0.70-1.12) |  |
|  | 6-10 |  | 51 (1.2) | 624 (1.4) | 0.79 (0.59-1.07) |  |
|  | 11-15 |  | 22 (0.5) | 310 (0.7) | 0.69 (0.44-1.07) |  |
|  | >15 |  | 14 (0.3) | 184 (0.4) | 0.75 (0.43-1.30) |  |
|  | p value for trend | |  |  |  | **0.016** |
|  |  |  |  |  |  |  |
|  | Other antifungals | |  |  |  |  |
|  | no prescription |  | 4231 (95.7) | 42443 (96.0) | 1.00 (reference) |  |
|  | 1-5 |  | 99 (2.2) | 797 (1.8) | **1.23 (1.00-1.52)** |  |
|  | 6-10 |  | 58 (1.3) | 526 (1.2) | 1.10 (0.83-1.45) |  |
|  | 11-15 |  | 21 (0.5) | 307 (0.7) | 0.68 (0.43-1.06) |  |
|  | >15 |  | 14 (0.3) | 157 (0.4) | 0.52 (0.36-0.75) |  |
|  | p value for trend | |  |  |  | 0.588 |
|  |  |  |  |  |  |  |
| **Topical antimicrobial drugs** | |  |  |  |  |  |
|  | Topical antibiotics | | |  |  |  |
|  | no prescription |  | 1889 (42.7) | 19648 (44.4) | 1.00 (reference) |  |
|  | 1-5 |  | 851 (19.2) | 7938 (18.0) | **1.11 (1.01-1.21)** |  |
|  | 6-10 |  | 798 (18.0) | 7764 (17.6) | 1.06 (0.97-1.16) |  |
|  | 11-15 |  | 501 (11.3) | 5091 (11.5) | 1.02 (0.91-1.14) |  |
|  | >15 |  | 384 (8.7) | 3789 (8.6) | 1.04 (0.90-1.21) |  |
|  | p value for trend | |  |  |  | 0.538 |
|  |  |  |  |  |  |  |
|  | Topical antifungals | | |  |  |  |
|  | no prescription |  | 2571 (58.1) | 26034 (58.9) | 1.00 (reference) |  |
|  | 1-5 |  | 625 (14.1) | 6208 (14.0) | 1.00 (0.91-1.10) |  |
|  | 6-10 |  | 573 (13.0) | 5494 (12.4) | 1.05 (0.95-1.16) |  |
|  | 11-15 |  | 342 (7.7) | 3611 (8.2) | 0.95 (0.84-1.09) |  |
|  | >15 |  | 312 (7.1) | 2883 (6.5) | 1.11 (0.96-1.30) |  |
|  | p value for trend | |  |  |  | 0.382 |
|  |  |  |  |  |  |  |
|  | Topical antivirals | | |  |  |  |
|  | no prescription |  | 4218 (95.4) | 42499 (96.1) | 1.00 (reference) |  |
|  | 1-5 |  | 62 (1.4) | 512 (1.2) | 1.19 (0.91-1.56) |  |
|  | 6-10 |  | 66 (1.5) | 513 (1.2) | 1.29 (0.99-1.67) |  |
|  | 11-15 |  | 43 (1.0) | 365 (0.8) | 1.16 (0.84-1.60) |  |
|  | >15 |  | 34 (0.8) | 341 (0.8) | 1.02 (0.71-1.46) |  |
|  | p value for trend | |  |  |  | 0.183 |
|  |  |  |  |  |  |  |
| adjusted for: | BMI, smoking, diabetes, congestive heart failure, myocardial, infarction, deep vein | | | | | |
|  | thrombosis, epilepsy, renal disease, opioid use, and infectious diseases | | | | | |
|  |  | | | | | |
|  | We performed Bonferroni correction for multiple testing of p value thresholds. Significant results after Bonferroni correction are indicated by an asterisk (*). | | | | | |
